# Clinical significance of serum PSA in breast cancer patients

**DOI:** 10.1186/s12885-019-6256-2

**Published:** 2019-10-29

**Authors:** Toru Hanamura, Koichi Ohno, Shinya Hokibara, Hideki Murasawa, Toshitsugu Nakamura, Hidehiko Watanabe, Machiko Kaizuka, Shinji Sawano, Hiroshi Koyama, Ken-ichi Ito

**Affiliations:** 1 Department of Breast and Endocrine Surgery, Suwa Red Cross Hospital, 5-11-50, Kogan-dori, Suwa, Nagano, 392-8510 Japan; 2 Division of Breast and Endocrine Surgery, Department of Surgery, Shinshu University School of Medicine, 3-1-1 Asahi, Matsumoto, Nagano, 390-8621 Japan; 3 Department of Pathology, University of Colorado Anschutz Medical Campus, 12800 East 19th Avenue, Aurora, CO 80045 USA; 4 Department of Diagnostic Pathology, Suwa Red Cross Hospital, 5-11-50, Kogan-dori, Suwa, Nagano, 392-8510 Japan; 5 Department of Laboratory Medicine, Suwa Red Cross Hospital, 5-11-50, Kogan-dori, Suwa, Nagano, 392-8510 Japan; 6 Medical checkup Center, Suwa Red Cross Hospital, 5-11-50, Kogan-dori, Suwa, Nagano, 392-8510 Japan; 7 Department of Surgery, Suwa Central Hospital, 4300, Tamagawa, Chino, Nagano, 391-8503 Japan; 8 Department of Surgery, Okaya City Hospital, 4-11-33, Hon-machi, Okaya, Nagano, 394-8512 Japan; 9 Koyama Breast and Thyroid Clinic, 1-2557-1, Jonan, Suwa, Nagano, 392-0017 Japan

**Keywords:** Breast cancer, Androgen signal, Androgen receptor, PSA

## Abstract

**Background:**

Recent preclinical data suggest that androgen receptor (AR) signaling plays a significant role in subsets of breast cancer. Clinical trials testing AR-targeting therapies in breast cancer have been conducted. Assessment of AR-signal in breast cancer tissue maybe useful for treatment selections. Prostate specific antigen (PSA) is the product of an androgen-responsive gene. Serum PSA (sPSA) can be detected in women by a highly sensitive assay although the concentration is much lower than that observed in males. We investigated if sPSA reflects tumor biology, including AR signaling in breast cancer patients.

**Methods:**

In this study, 132 healthy controls and 144 breast cancer patients were enrolled. sPSA was evaluated by the chemiluminescent enzyme immunoassay (CLEIA) method. Correlations between sPSA and the various clinicopathological factors were analyzed.

**Results:**

In post-menopausal state, sPSA detection rate was significantly higher in breast cancer patients compared with controls (27.4% vs 11.3%: *p* = 0.0090), but not in the whole cohort (29.2% vs 25.8%: *p* = 0.5265) or pre-menopausal subgroup (37.0% vs 42.6%: *p* = 0.6231). In post-menopausal breast cancer cases, higher sPSA value was associated with clinic-pathological factors including the expression of AR protein in primary legion. In a correlation analysis of quantitative data limited to post-menopausal metastatic breast cancer (MBC), sPSA was positively, albeit weakly correlated with clinic-pathological features including serum testosterone levels and AR positivity.

**Conclusions:**

Our data suggest that sPSA may reflect tumor biological properties including AR activity in post-menopausal breast cancer.

## Background

Breast cancer is the most common malignancy in women worldwide and one of the leading causes of cancer death. While specific therapeutics have been developed and treatment outcomes have improved, about a third of patients treated for apparently localized breast cancer develop metastatic disease [1–3]. Therefore, it is necessary to further improve the outcome of initial treatment and to develop more effective treatment strategies for recurrent, metastatic disease.

The majority of breast cancers are hormone-dependent and estrogen deprivation therapy is the major treatment strategy [1, 2]. Although in the adjuvant setting, women can be treated with selective estrogen receptor modulators (SERMs) or aromatase inhibitors (AIs), some patients exhibit de novo resistance and some develop acquired resistance over time [1–4]. Recently, to model AI-resistant breast cancer we generated variant cell lines from the estrogen receptor (ER)-positive T-47D breast carcinoma cell line under estrogen-depleted, excess androgen conditions. These variant cell lines had increased androgen receptor (AR) and exhibited decreased expression of ER and no growth response to estrogen. Furthermore, androgen markedly induced proliferation in these cell lines [5–7]. In another study, AR overexpression led to tamoxifen resistance in in vitro models of breast cancer, implicating the involvement of AR signaling in tamoxifen resistance [8]. Therefore, it is hypothesized that a possible resistance mechanism could be tumor adaptation from ER dependence to AR dependence [4, 9–11]. AR-targeting therapies for ER-positive breast cancer (NCT02910050 for bicalutamide, NCT01597193, NCT02955394, NCT02953860, NCT02007512 for enzalutamide, respectively) are currently being conducted.

AR dependency has also been suggested in a subset of ER-negative, AR-positive breast cancers [12–15]. Triple negative breast cancer (TNBC) is defined by the lack of estrogen and progesterone receptors as well as an absence of HER2 (human epidermal growth factor receptor 2) amplification. Because of the lack of specific targeted therapy, 30–40% of patients with early-stage TNBC develop metastatic disease and succumb to the cancer, despite receiving standard multi-agent adjuvant chemotherapy [16, 17]. Both molecular and immunohistochemical analyses demonstrate that a subset of TNBC expresses AR. Recently, numerous preclinical studies have validated the use of AR modulation in limiting cell proliferation, growth on soft agar, and tumor initiation in vivo [14, 15, 18–20] and there are ongoing clinical trials evaluating the efficacy of AR antagonists in ER-negative breast cancer (NCT00468715, NCT03055312, NCT03090165, NCT02605486 for bicalutamide, NCT02750358, NCT02689427, NCT01889238, NCT02457910 for enzalutamide, respectively).

AR functions as a transcription factor upon binding to androgen, and regulates the transcription of target genes [21]. Because AR signaling plays pivotal roles in prostate cancer, AR targeting therapies are widely used for prostate cancer treatment [22]. Prostate specific antigen (PSA) is a serine protease encoded in humans by the kallilrein related peptidase 3 (*KLK3*) gene [23]. The transcription of the *KLK3* gene is positively regulated by AR [21]. Therefore, PSA is one of the most widely used serum biomarkers for the diagnosis and follow-up of prostate cancer [24]. Although widely thought to be exclusively produced in prostate gland [25], extra-prostatic production of PSA has been reported in various conditions including normal breast tissue and benign and malignant breast tumors [26]. Furthermore, it was reported that serum PSA (sPSA) can be detected in breast cancer patients by highly sensitive assay [23, 27, 28]. If sPSA levels reflect the amount of AR signaling or AR dependency of the tumor in breast cancer patient, it may be useful for effective treatment selection. However, its biological significance in relation to breast cancer has not been established.

In this study, we investigated whether sPSA might reflect tumor biology, including AR signaling. Using blood samples from both healthy controls and breast cancer patients, individuals were enrolled regardless of age, clinicopathological factor or treatment history. sPSA was evaluated by chemiluminescent enzyme immunoassay (CLEIA) method at various timepoints for each case. Then correlations between sPSA and clinicopathological factors were analyzed.

## Methods

This study was conducted in Suwa Red Cross Hospital, Suwa Central Hospital, Okaya City Hospital and Koyama Clinic during August 2017 to January 2018. All procedures performed in this study involving human participants were conducted with approval of the Suwa Red Cross Hospital ethics committee (reference number: 29–40) in accordance with the ethical standards of the institutional research committee and with the 1964 Helsinki declaration and its later amendments. Written informed consent was obtained from all participants for protocols including blood collection, reviewing case records and use of archival samples.

### Subjects

Breast cancer patients, with the exception of cases without relapse after surgery, were enrolled regardless of age, clinicopathological factor or treatment history. Pre-operative stage 0 - III, de novo stage IV and recurrent breast cancer cases were included. Healthy women who performed a mammography examination in annual general checkup were enrolled as healthy controls. Due to the short period of the study, it was not possible to match breast cancer patients and healthy controls by age or menopause status. It is reported that sPSA may show higher values in benign breast disease including mammary cysts and fibroadenoma [26]. However, since the purpose of this analysis was not to verify whether sPSA is a marker for breast cancer detection or discrimination of malignancy from benign breast disease, but to determine whether sPSA reflects breast cancer biological characteristics, patients with benign breast disease were excluded from this analysis. Women with current morbidity or history of uterine fibroids, polycystic ovary syndrome, benign ovarian tumor, hirsutism, malignancy other than breast cancer, use of oral contraceptive and hormone replacement therapy were also excluded regardless of breast cancer group or healthy control group in this study, because these diseases are reported to have higher sPSA values [26]. Breast cancer patients with only ipsilateral axillary recurrence or loco-regional recurrence were excluded. Women who had any abnormality in mammography were excluded from healthy controls.

### Data collection

Clinical data including age, menopausal state, clinical stage, disease status and treatment history were collected by reviewing patient case records. At the time of blood sample collection, subjects who had amenorrheic for more than 1 year were defined as postmenopausal, whether this was natural or post-chemotherapy. All other subjects were defined as premenopausal. Clinical stage was assessed based on UICC TNM classification [29]. Anastrozole, letrozole, and exemestane were defined as AIs, tamoxifen and toremifene as SERMs, and fulvestrant as selective estrogen receptor degrader (SERD). Recurrence during adjuvant endocrine therapy or within 12 months after completion of adjuvant endocrine therapy and disease progression during treatment for metastatic disease were defined as drug resistant.

### Blood samples

Blood samples from Stage 0 - III breast cancer patients other than patient who underwent pre-operative adjuvant chemotherapy were obtained within one month before surgery for primary lesion (*n* = 62). Blood samples from patients who underwent pre-operative adjuvant chemotherapy (*n* = 5) were obtained after the core needle biopsy of the primary lesion within one month before starting the chemotherapy. Blood samples from MBC including de novo stage IV and recurrent breast cancer patients were obtained before starting treatment (*n* = 12) or on treatment (*n* = 65) for metastatic disease. Blood samples were collected by venipuncture in a plain plastic tube. After centrifugation at 2000×g for 7 min; the sera were stored frozen (− 80 °C) until analysis. At the start of the study, a pilot study was conducted for ten samples using five sPSA testing kits, and the kit which showed highest sPSA detection rate was selected for further study (Additional file 1 Table S1). Sandwich-type CLEIA was performed for serum total PSA quantitation according to the manufacturer’s standard protocol using TOSOH PSA kit AIA pack CL (TOSOH CO., LTD., Tokyo, Japan). Briefly, the monoclonal antibody against PSA was immobilized on a microtiter plate and serum samples were added. After incubation at RT, the alkaline phosphatase-linked secondary antibody for PSA was added. After another incubation, DIFURAT® was added as substrate. Chemiluminescent was detected by the automated AIA®-CL2400 platform ((TOSOH CO., LTD., Tokyo, Japan). The detection limit of immunoassays is 3 ng/L. Intra-assay and inter-assay CVs are 2.0–3.1% and 3.2–3.7%, respectively. For quantitation of serum estradiol and testosterone the competitive-type electrochemiluminescence immunoassay was performed according to the manufacturer’s standard protocol using an Elecsys® Estradiol IV test kit and an Elecsys® testosterone II test kit (Roche, Basel, Switzerland), respectively. The detection limits for estradiol and testosterone are 5 pg/mL and 0.025 ng/mL, respectively. With regard to testing for sPSA, estradiol and testosterone, samples showing the value under the detection limit of each test were considered as inferior as this value, non-parametric tests were performed during statistical analysis including these test values. Values of 0 in the graph represent samples below the detection limit.

### Tumor samples

Tumor samples from breast cancer patients, other than de novo stage IV, were obtained during surgery for primary lesion. In the case of de novo stage IV tumors, specimens were collected from the core needle biopsy of the primary tumor. All specimens were fixed with 10% formalin and embedded in paraffin wax.

Pathological data including histological type, ER / PgR / HER2 / Ki67 status and nuclear grade were collected by reviewing patient case records. ER, PgR, and HER2 statuses were evaluated by IHC staining. The cut-off value for ER and PgR positivity was set at ≥1% [30]. Tumors were considered to overexpress HER2 if they were given a score of 3 following IHC staining, or if they showed ≥2.0-fold amplification of the *HER2* gene, as assessed by fluorescence in situ hybridization (FISH). FISH testing was only performed for tumors that scored 2 during IHC staining [31]. The cut-off value of Ki67 was set at 20% in this study [32]. The nuclear grade composed of nuclear atypia and mitotic counts were evaluated based on the Japanese Classification of Breast Cancer [33, 34].

Expression of AR and PSA in primary lesion was evaluated by IHC method using archival samples. Mouse monoclonal antibodies for AR (clone AR441) and PSA (clone 35H9) were purchased from Agilent Technologies (Santa Clara, CA, USA) and Leica Biosystems (Wetzlar, Germany), respectively. The IHC staining was performed using the Ventana Benchmark LT automated IHC device (Roche Diagnostics, Basel, Switzerland) and the reaction product was detected with Ventana iVIEW DAB Universal kit (Roche Diagnostics). The antigen-antibody complex was visualized with diaminobenzidine (DAB) and counter-stained with hematoxylin. AR immunoreactivity was detected in the nuclei of breast carcinoma cells, and the percentage of immunoreactive cells, i.e., labeling index (LI), was determined [18]. The median of AR LI, 20%, was taken as the cut-off value for the AR expression. PSA immunoreactivity was considered positive if any cytoplasmic staining was observed in the carcinoma cells [5].

### Statistical analysis

Statistical analyses were performed using the StatFlex 6.0 software program (Artech Co., Ltd., Osaka, Japan). In comparison between groups, sPSA-detected cases and non-detected cases were defined as sPSA positive and negative, respectively. Chi-squared test was used for comparison of these group. Spearman’s rank correlation coefficient was applied for correlation between quantitative data and sPSA values using absolute values of sPSA but, for visualization, log-transformed values of sPSA were used for the graphs. Representative values of sPSA were shown in Median (Inter-quartile range). Mann-Whitney *U* test or Kruskal Wallis test were used for comparison of sPSA among two group or multiple groups respectively. Samples with unknown values were excluded from the statistical analysis. Values of *P* < 0.05 were considered statistically significant. Actual *p* values are shown in figures for all of statistical testing.

## Results

### sPSA value in breast cancer patients and healthy controls

**Fig. 1:**
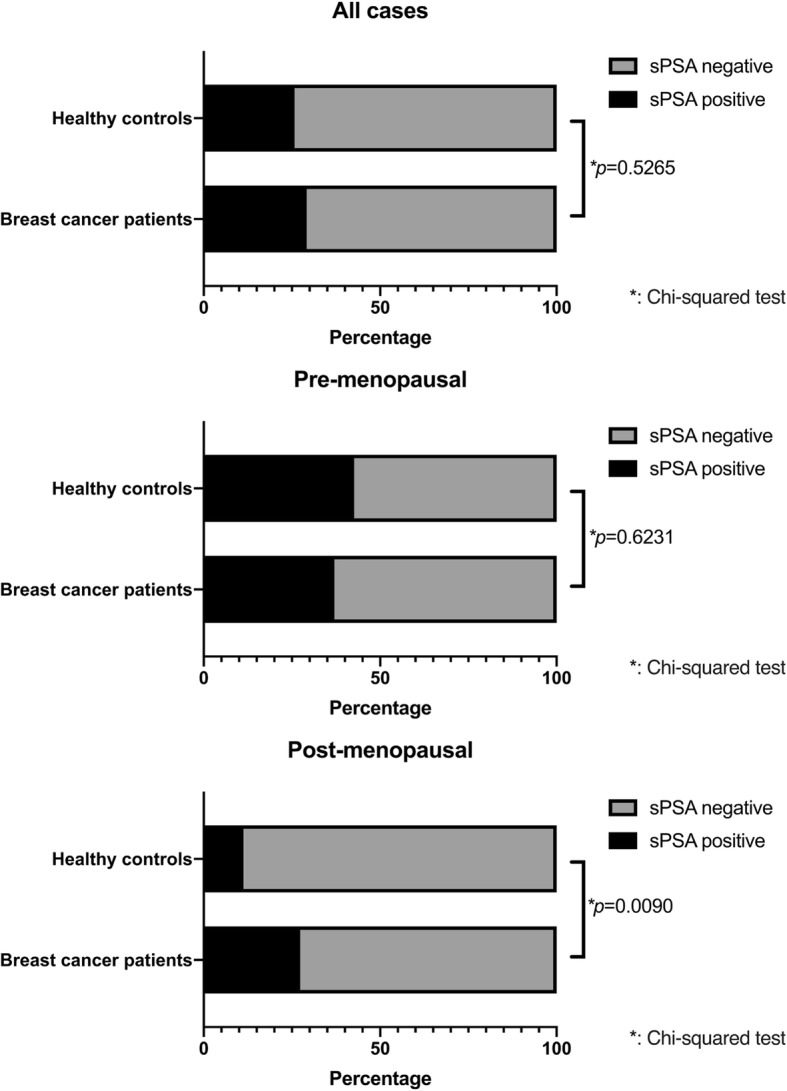
Serum PSA detection rate in breast cancer patients and healthy controls (*n* = 276). The X axis shows sPSA detection rate. The difference between two groups were analyzed by Chi-squared test. Values of *p* < 0.05 were considered statistically significant

In present study, 132 healthy controls (53.8% were postmenopausal) and 144 breast cancer patients (81.3% were postmenopausal) were enrolled. Characteristics of breast cancer cases and healthy controls are shown in Table 1. sPSA was detected in 29.2 and 25.8% of breast cancer patients and controls, respectively, with no statistically significant difference between groups. Similarly, there was no statistically significant difference in the levels of sPSA between breast cancer patients and controls (0 [0–4.0] ng/L vs 0 [0–3.0] ng/L; *p* = 0.3409). In subsequent analyses sPSA values from pre- and post-menopause subjects were separated. In pre-menopausal state, there was no significant difference in sPSA detection rate between breast cancer patients and controls (37.0% vs 42.6%: *p =* 0.6231). However, in the post-menopausal state sPSA detection rate was significantly higher in breast cancer patients compared with controls (27.4% vs 11.3%: *p* = 0.0090). (Fig. 1, Table 2). Similar results were also obtained in the comparison of the levels of sPSA (Additional file 1 Table S2).

**Table 1:** Clinical and histopathological characteristics of 132 healthy controls and 144 breast cancer patients

		Healthy control (*n* = 132)	Breast cancer (*n* = 144)
		No of cases (%)	No of cases (%)
Age (mean ± *SD*)		53.1 ± 10.7	62.9 ± 13.2
Menopausal status	Pre-menopausal	61 (46.2)	27 (18.8)
	Post-menopausal	71 (53.8)	117 (81.3)
sPSA detection rate		25.8%	29.2%
sPSA ng/l (Median [IQR^*^])		0 (0–3)	0 (0–4)
Clinical stage	Non-MBC; Stage 0-III	–	67 (46.5)
	MBC; Stage IV, Recurrence	–	77 (53.5)
Histological type	Invasive ductal carcinoma	–	102 (70.8)
	Ductal carcinoma in situ	–	9 (6.3)
	Invasive lobular carcinoma	–	11 (7.6)
	Lobular carcinoma in situ	–	0 (0)
	Special type	–	22 (15.3)
Subtype	Luminal; ER+ / HER2-	–	99 (68.8)
	Luminal HER2; ER+ / HER2+	–	16 (11.1)
	HER2 enriched; ER- / HER2+	–	8 (5.6)
	TNBC; ER- / HER2-	–	21 (14.6)
Histological grade	1	–	82 (56.9)
	2	–	40 (27.8)
	3	–	22 (15.3)
Ki67 positivity	< 20%	–	60 (41.7)
	≧20%	–	81 (56.3)
	Unknown	–	3 (2.1)

*: inter-quartile range

**Table 2:** sPSA detection rate in breast cancer patients and healthy controls

	sPSA positive (%)	sPSA negative (%)	*p* value
All cases (n = 276)			0.5265
Healthy control	34 (25.8)	98 (74.2)	
Breast cancer	42 (29.2)	102 (70.8)	
Pre-menopausal cases (*n* = 88)			0.6231
Healthy control	26 (42.6)	35 (57.4)	
Breast cancer	10 (37.0)	17 (63.0)	
Post-menopausal cases (*n* = 188)			0.0090
Healthy control	8 (11.3)	63 (88.7)	
Breast cancer	32 (27.4)	85 (72.6)	

### sPSA value in post-menopausal breast cancer patients

In analysis limited to post-menopausal breast cancer cases, sPSA detection rate was significantly higher in MBC compared with non-MBC (36.1% vs 13.3%: *p* = 0.0072). Similarly, sPSA detection rate was significantly higher in high AR (≥ 20%) cases compared with low AR (< 20%) cases (39.0% vs 14.5%: *p* = 0.0034). sPSA detection rate was higher in low Ki67 (< 20%) cases compared with high Ki67 (≥ 20%) (36.5% vs 19.4%: *p* = 0.0400). There was no significant difference in sPSA detection rate due to histological type, tumor subtype, PSA expression by IHC and nuclear grade in primary lesion (Table 3). Similar results were also obtained in the comparison of the levels of sPSA (Additional file 1 Table S3).

**Table 3:** sPSA detection rate in post-menopausal breast cancer patients (n = 117)

	n	sPSA positive (%)	sPSA negative (%)	*p* value
Clinical stage				0.0072
Non-MBC; Stage 0-III	45	6 (13.3)	39 (86.7)	
MBC; Stage VI, Recurrence	72	26 (36.1)	46 (63.9)	
Histological type				0.9320
Invasive ductal carcinoma	82	21 (25.6)	61 (74.4)	
Ductal carcinoma in situ	6	2 (33.3)	4 (66.7)	
Invasive lobular carcinoma	10	3 (30.0)	7 (70.0)	
Special type	19	6 (31.6)	13 (68.4)	
Subtype				0.3028
Luminal; ER+ / HER2-	83	19 (22.9)	64 (77.1)	
Luminal HER2; ER+ / HER2+	13	6 (46.2)	7 (53.8)	
HER2 enriched; ER- / HER2+	7	2 (28.6)	5 (71.4)	
TNBC; ER- / HER2-	14	5 (35.7)	9 (64.3)	
Androgen receptor				0.0034
< 20%	55	8 (14.5)	47 (85.5)	
≥ 20%	59	23 (39.0)	36 (61.0)	
Unknown	3	1 (33.3)	2 (66.7)	
PSA (IHC of primary lesion)				0.1271
Positive	64	21 (32.8)	43 (67.2)	
Negative	50	10 (20.0)	40 (80.0)	
Unknown	3	1 (33.3)	2 (66.7)	
Nuclear grade				0.7405
1	67	18 (26.9)	49 (73.1)	
2	36	9 (25.0)	27 (75.0)	
3	14	5 (35.7)	9 (64.3)	
Ki67 (LI)				0.0400
< 20%	52	19 (36.5)	33 (63.5)	
≥ 20%	62	12 (19.4)	50 (80.6)	
Unknown	3	1 (33.3)	2 (66.7)	

### Correlation between sPSA and various clinicopathological factors in post-menopausal MBC

**Fig. 2:**
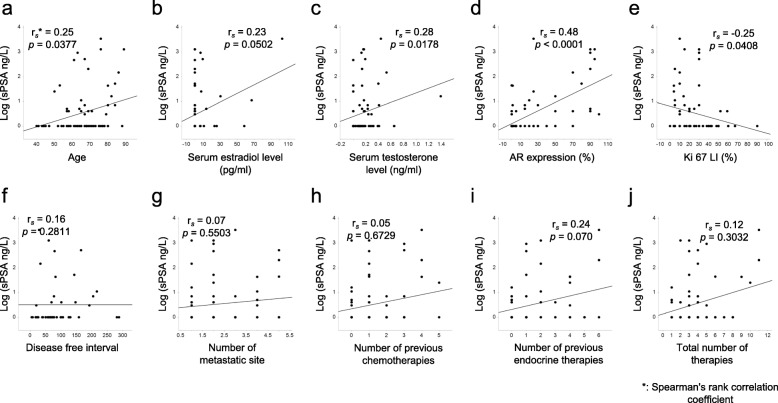
Correlation between sPSA and various clinicopathological factors in post-menopausal MBC (*n* = 72). The vertical axis shows Log conversion of the sPSA value. Lines in the graph indicate the regression line. The relationship between these two values was analyzed by Pearson’s correlation. Values of *p* < 0.05 were considered statistically significant. Actual *p* values are shown in the figures when the *p* value was between 0.05 and 0.10. Values of *p* > 0.10 are shown in figures as not significant (NS)

We performed a correlation analysis of various quantitative data and sPSA in post-menopausal MBC cases, since these patients showed high sPSA values in the above analysis suggesting that sPSA of these patients are more likely to be derived from breast cancer tissue. In these patients, sPSA was weakly but positively correlated with age (rS = 0.25, *p =* 0.0377), serum testosterone levels (ng/ml) (rS = 0.28, *p =* 0.0178) and AR positivity (%) (rS = 0.48 *p <* 0.0001). Likewise, sPSA was negatively correlated with Ki67 (rS = − 0.25, *p =* 0.0178). sPSA did not correlate with the serum estrogen level, disease free interval, number of metastatic organs, number of previous chemotherapies the number of previous endocrine therapies or total number of therapies (Fig. 2).

### Difference in sPSA value due to previous endocrine therapy

**Fig. 3:**
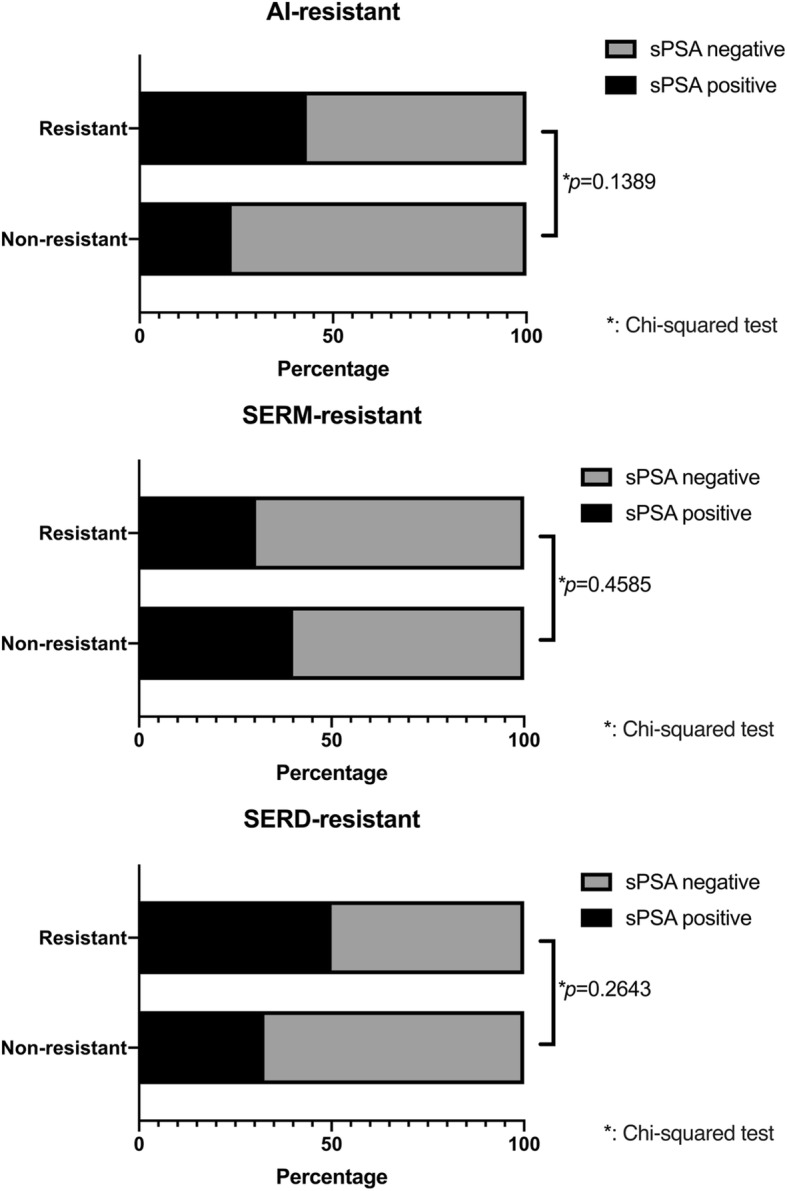
Difference in sPSA values due to previous endocrine therapy (*n* = 58). The X axis shows sPSA detection rate. The difference between two groups were analyzed by Chi-squared test. Values of *p* < 0.05 were considered statistically significant

**Fig. 4:**
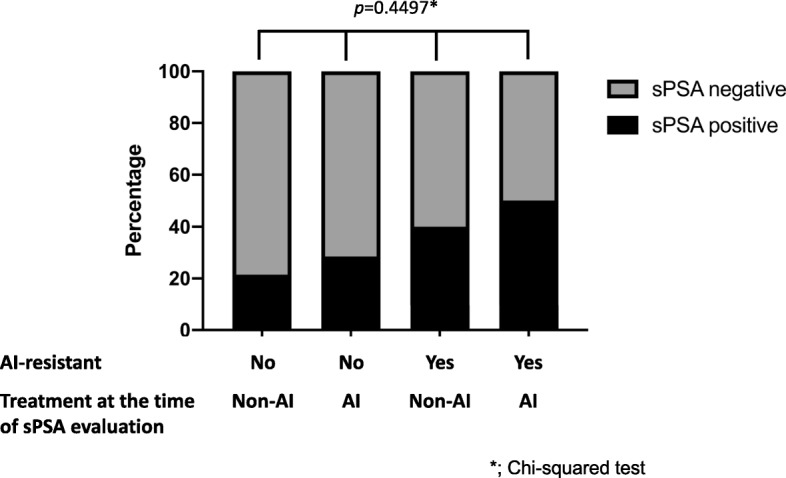
Difference in sPSA values due to treatment and AI resistance property (n = 58). The vertical axis shows sPSA detection rate. Chi-squared tests were used for comparison of sPSA detection rate among multiple groups. Values of *p* < 0.05 were considered statistically significant

In the analysis limited to post-menopausal ER positive MBC, although there was no statistical difference in sPSA detection rate due to resistance to AIs, SERMs or SERDs (Fig. 3, Table 4), AI-resistant cases have significantly higher sPSA levels compared with non-AI resistant cases (0 [0–29.5] ng/L vs 0 [0–1.0] ng/L; *p* = 0.0473) (Additional file 1 Table S4). Although there were no statistically significant differences, sPSA detection rate and their levels tended to be higher in AI-resistant cases compared with non-AI resistant cases, regardless of whether they were on AI therapy at the time of the blood sample collection (Fig. 4 and Additional file 2 Figure S1).

**Table 4:** Difference in sPSA detection rate due to previous endocrine therapy (n = 58)

	n	sPSA positive (%)	sPSA negative (%)	*p* value
Aromatase inhibitor resistance				0.1389
Yes	37	16 (43.2)	21 (56.8)	
No	21	5 (23.8)	16 (76.2)	
SERM resistance				0.4584
Yes	23	7 (30.4)	16 (69.6)	
No	35	14 (40.0)	21 (60.0)	
SERD resistance				0.2643
Yes	12	6 (50.0)	6 (50.0)	
No	46	15 (32.6)	31 (67.4)

## Discussion

As mentioned above, the recent use of ultrasensitive PSA immunoassays has enabled detection of PSA in normal female serum, even if at extremely low concentrations compared with that of males [35]. In the context of breast cancer, sPSA was reported to be higher in breast cancer patients compared with healthy control and decreased in the serum of breast cancer patients after surgery [27, 28] indicating that PSA derived from breast cancer tissues can be detected in serum. In our study, the above findings indicate that under normal physiological conditions sPSA was detectable before menopause and is low to non-detectable following menopause (Fig. 1, Table 2). Notably, in breast cancer patients sPSA was detected after menopause, which suggests that sPSA in these post-menopausal patients may be from the tumor itself. However, in this study there were no significant difference in sPSA between breast cancer patients and normal control in the global analysis except for post-menopausal women. This differs from previous reports showing the higher sPSA levels in both of pre- and post-menopausal breast cancer patients compared with control [36]. This may be due to the relatively few premenopausal cases. Therefore, further investigation is needed. Subsequent analyses were done only for the post-menopausal case. In addition, in the previous study [27, 28], sPSA levels are associated with younger age, premenopausal status regardless of health condition, and larger tumor size in breast cancer cases, which corresponds well with our results that sPSA was higher in the pre-menopausal state and in advanced disease, such as MBC (Fig. 1, Tables 2, 3). Although, in our study, sPSA values were under the detection limit in more than 70% of samples and showed large data deviation, similar trends were found in the other reports from Black et al. [27]. Existing research about sPSA in breast cancer had focused on its diagnostic value [27, 28, 36, 37]. Its correlation to biological features associated with breast cancer have not been fully established, especially in the context of the association of androgens or AR signaling. Therefore, this is the first study which analyzed in detail the relationships between sPSA and various biological characteristics of breast cancer and, in particular, the relation to serum androgen level and AR expression in the primary tumor tissue.

In our analysis limited to post-menopausal breast cancer cases, sPSA values were significantly higher in MBC (de novo stage IV and recurrence) compared with non-MBC (stage 0 – III) (Table 3). This seems to reflect the tumor volume rather than biological characteristics of the tumor. Black et al. reported that sPSA was significantly associated with larger breast tumor size [27]. The most interesting result was that sPSA was positively correlated with serum testosterone levels and AR positivity in post-menopausal MBC (Fig. 2) suggesting that sPSA might function as a readout of AR activity in tumors. On the contrary, tissue PSA expression in the primary tumor did not correlate with sPSA levels (Table 3). This may be because many cases were treated prior to blood sample collection, so tumor biological features might have changed with treatment. The evaluation of PSA by IHC in breast cancer has not been fully established and positive rates of PSA vary greatly depending on reports [5, 38, 39]. It may be useful to verify by combining quantitative methods such as time resolved immunofluorometric assay or Mass Spectrometry-Based Proteomic Profiling [40, 41]. In the present study, we found a negative correlation between sPSA and Ki67 in post-menopausal MBC (Table 3, Fig. 2). In the majority of studies, AR expression in ER-positive tumors or TNBCs has been associated with favorable characteristics including lower Ki67 positivity [42–44]. Therefore, assuming that sPSA reflects the function of AR in the tumor, this result is consistent with our result. Next, we focused on sPSA values in various endocrine therapy-resistant breast cancers. As mentioned above, it has been suggested that tumors may shift their dependence from ER to AR as a possible endocrine therapy resistance mechanism [4, 9–11]. We hypothesized that if sPSA acts as a readout of AR signaling in tumors, then sPSA levels might change during endocrine therapy. Therefore, we compared sPSA levels in patients with resistance to endocrine therapies. It is known that the androgen concentration in the tumor can increase with AI treatment [45]. However, in our analysis, sPSA tended to be higher in AI-resistant cases compared with non-AI resistant cases, regardless of whether patients were on AI therapy at the time of the blood sample collection (Fig. 4. Additional file 1 Figure S1). These findings suggest that elevation of sPSA was not simply caused by increase in androgen following AI treatment and that some of the ER-positive post-menopausal MBC may switch from ER-dependent to AR-dependent as a mechanism of resistance to traditional endocrine therapies, particularly AI. All of the above results show that sPSA may reflect tumor biological properties, including androgen signals and changes associated with treatment in post-menopausal breast cancer.

In metastatic or recurrent breast cancer, treatment selection is made based on the biological information obtained from primary lesion; however, after treatment, tumor biology evolves during the course of treatment. However, it is difficult to take a biopsy of metastatic lesions frequently. Serum PSA can be assessed by blood exam which is a minimally invasive examination. Based on the result of present study, we hypothesize that PSA may be useful for effective treatment selection, especially for AR-targeting therapies in post-menopausal breast cancer patients. However, because this analysis is an observational study, it is difficult to verify whether sPSA reflects AR signal of the tumor in a strict sense. Therefore, further analysis using in-vitro and in-vivo models, including interventional clinical studies using AR-targeted therapies, should be performed.

## Conclusion

Tumor derived sPSA was detectable in a portion of post-menopausal breast cancer patients (27.4%). Serum PSA levels were weakly associated with serum testosterone levels and AR positivity in primary tumors suggesting that sPSA may reflect some tumor biological properties including androgen signals in post-menopausal breast cancer. Thus, serum PSA may be useful for identifying patients with tumors expressing active AR.

## Supplementary information

**Additional file 1: Table S1.** Preliminary experiment for selection of sPSA measurement kit (*n* = 10). **Table S2.** sPSA values in breast cancer patients and healthy controls. **Table S3.** sPSA values in post-menopausal breast cancer patients (*n* = 117). **Table S4.** Difference in sPSA values due to previous endocrine therapy (*n* = 58).

**Additional file 2: Figure S1. (PDF 42 kb)**

## Data Availability

The datasets analyzed during the current study are not publicly available due to no suitable repository but are available from the corresponding author on reasonable request.

## Abbreviations

AI: aromatase inhibitor
AR: androgen receptor
CLEIA: chemiluminescent enzyme immunoassay
ER: estrogen receptor α
FISH: fluorescence in situ hybridization
HER2: human epidermal growth factor receptor 2
KLK3: kallikrein related peptidase 3
MBC: metastatic breast cancer
PgR: progesterone receptor
PSA: prostate specific antigen
SERDs: Selective estrogen receptor degraders
SERMs: Selective estrogen receptor modulators
sPSA: serum PSA
TNBC: Triple negative breast cancer
