# Supplementary material for: Clinical significance of serum PSA in breast cancer patients
Source: BMC Cancer. 2019 Oct 29;19:1021. doi: 10.1186/s12885-019-6256-2 (PMC6819570; doi:10.1186/s12885-019-6256-2)
Supplement: Supplementary file 1 — Additional file 1: Table S1. Preliminary experiment for selection of sPSA measurement kit (n = 10). Table S2. sPSA values in breast cancer patients and healthy controls. Table S3. sPSA values in post-menopausal breast cancer patients (n = 117). Table S4. Difference in sPSA values due to previous endocrine therapy (n = 58). [file 12885_2019_6256_MOESM1_ESM.doc]

table S1. Preliminary experiment for selection of sPSA measurement kit (n=10).

|  | TOSOH PSA kit AIA pack CL | | Kit A | Kit B | Kit C | Kit D |
| --- | --- | --- | --- | --- | --- | --- |
| Detection limit | 0.003ng/ml〜 | 0.002ng/ml〜 | | 0.008ng/ml〜 | 0.01ng/ml〜 | 0.8ng/ml〜 |
| #1 | 0.025 | 0.024 | | 0.022 | 0.020 | No Sample |
| #2 | ND* | ND | | ND | ND | No Sample |
| #3 | 0.006 | 0.011 | | 0.013 | ND | No Sample |
| #4 | ND | ND | | ND | ND | ND |
| #5 | ND | ND | | ND | ND | ND |
| #6 | ND | ND | | ND | ND | ND |
| #7 | 0.060 | ND | | ND | ND | ND |
| #8 | 0.009 | 0.007 | | 0.008 | ND | ND |
| #9 | ND | ND | | ND | ND | ND |
| #10 | ND | ND | | ND | ND | ND |

*: Not detectable

table S2. sPSA values in breast cancer patients and healthy controls.

|  |  | sPSA ng/L (Median [IQR^*^]) | | *p* value |
| --- | --- | --- | --- | --- |
|  |  | Healthy control | Breast cancer |  |
| All cases  (n = 276) |  | 0 (0 - 3.0)  (n=132) | 0 (0 - 4.0)  (n=144) | 0.3409 |
| Pre-menopausal cases  (n = 88) |  | 0 (0 - 6.0)  (n = 61) | 0 (0 - 5.0)  (n = 27) | 0.6222 |
| Post-menopausal cases  (n = 188) |  | 0 (0 - 0)  (n= 71) | 0 (0 - 4.0)  (n = 117) | 0.0045 |

*: inter-quartile range

table S3. sPSA values in post-menopausal breast cancer patients (n=117).

|  | n | PSA (ng/L);  median (IQR*) | *p* value  vs each category |
| --- | --- | --- | --- |
| Clinical stage |  |  | 0.0045 |
| Non-MBC; Stage 0-III | 45 | 0 (0 - 0) |  |
| MBC; Stage VI, Recurrence | 72 | 0 (0 - 5.0) |  |
| Histological type |  |  | 0.9310 |
| Invasive ductal carcinoma | 82 | 0 (0 - 3.0) |  |
| Ductal carcinoma *in situ* | 6 | 0 (0 - 4.0) |  |
| Invasive lobular carcinoma | 10 | 0 (0 - 3.0) |  |
| Special type | 19 | 0 (0 - 4.0) |  |
| Subtype |  |  | 0.3590 |
| Luminal; ER+ / HER2- | 83 | 0 (0 - 0) |  |
| Luminal HER2; ER+ / HER2+ | 13 | 0 (0 - 5.5) |  |
| HER2 enriched; ER- / HER2+ | 7 | 0 (0 - 3.0) |  |
| TNBC; ER- / HER2- | 14 | 0 (0 – 4.0) |  |
| Androgen receptor |  |  | 0.0019 |
| <20% | 55 | 0 (0 - 0) |  |
| ≥20％ | 59 | 0 (0 – 6.8) |  |
| Unknown | 3 | NA |  |
| PSA (IHC of primary lesion) |  |  | 0.1552 |
| Positive | 64 | 0 (0 - 4) |  |
| Negative | 50 | 0 (0 - 0) |  |
| Unknown | 3 | NA |  |
| Nuclear grade |  |  | 0.7305 |
| 1 | 67 | 0 (0 - 3.0) |  |
| 2 | 36 | 0 (0 - 2.0) |  |
| 3 | 14 | 0 (0 - 6.0) |  |
| Ki67 (LI) |  |  | 0.0340 |
| <20% | 52 | 0 (0 - 7.0) |  |
| ≥20％ | 62 | 0 (0 - 0) |  |
| Unknown | 3 | NA |  |
| *: inter-quartile range |  |  |  |

table S4. Difference in sPSA values due to previous endocrine therapy (n=58).

|  | n | PSA (ng/L);  median (IQR*) | *p* value  vs each category |
| --- | --- | --- | --- |
| Aromatase inhibitor resistance |  |  | 0.0473 |
| Yes | 37 | 0 (0 - 29.5) |  |
| No | 21 | 0 (0 – 1.0) |  |
| SERM resistance |  |  | 0.5985 |
| Yes | 23 | 0 (0 - 3.8) |  |
| No | 35 | 0 (0 – 7.0) |  |
| SERD resistance |  |  | 0.3048 |
| Yes | 12 | 1.5 (0 - 18.0) |  |
| No | 46 | 0 (0 – 5.0) |  |

*: inter-quartile range
