# Supplementary figures and images for: Clinical significance of serum PSA in breast cancer patients
Source: BMC Cancer. 2019 Oct 29;19:1021. doi: 10.1186/s12885-019-6256-2 (PMC6819570; doi:10.1186/s12885-019-6256-2)

## Supplementary figure 1

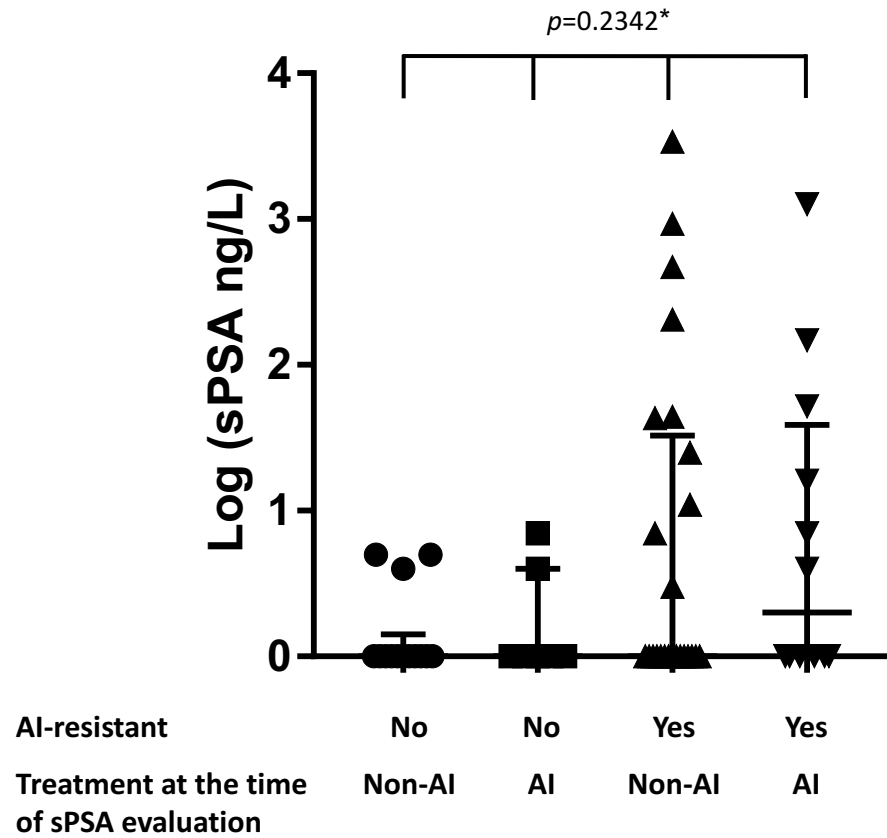

\*; Kruskal Wallis test

Supplement: Supplementary file 2 — Additional file 2: Figure S1. (PDF 42 kb) [file 12885_2019_6256_MOESM2_ESM.pdf]
